# Bubonic Plague

**Published:** 1897-01-09

**Authors:** 


### BUBONIC PLAGUE.

The lecture on Bubonic Plague, lately delivered before the Epidemiological Society and the discussion which followed it, have served to fix in the mind certain points, which it is well should be remembered, however little, it is to be hoped, we may personally have to deal with this disease.

Although the term "bubonic" has lately been applied to the disorder as a distinctive appellation, it must be admitted that the bubo is, in a considerable proportion of the cases, by no means a marked symptom. It seems, however, that a multiple adenitis is almost constantly discovered after death, even in the very cases in which no bubo could be discovered clinically. Dr. Cantlie defines the disease thus: an acute febrile disease of an intensely fatal nature, characterised by inflammation of the lymphatic glands, marked cerebral and vascular disturbances, and the presence of a specific bacillus.

The occurrence of the disease in the lower animals is a matter of considerable interest, which has been prominently brought out by recent observations, especially its association with rats.

Yersin has observed that flies, also, are attacked and killed by the disease—a matter of great importance in regard to its distribution. Cantlie states that one sub-family of the rat tribe, that of the Nesokia, reaches from Palestine to Formosa. This, he says, is the only animal which presents a habitation well nigh corresponding to the present distribution of plague.

A matter of still more interest is the observation that there exists a mild form of bubonic plague, the pestis minor, which rarely causes death, and for which but few people are confined to bed. This disease has been perfectly well recognised, but it has generally been considered to be distinct from true plague, although it has been noticed to precede or follow certain severe epidemics of the more serious disease. But the news now comes from Calcutta of the discovery of a bacillus, allied in every respect to the Kitasato bacillus of true plague, and assocated with the variety of plague known as pestis minor.

Dr. Patrick Manson, writing in the *Practitioner* on the same subject, says: "This discovery, if confirmed, is one of great importance; for. taken in conjunction with the historical fact alluded to—that epidemics of a similar innocent nature have culminated in outbreaks of malignant plague—it brings us face to face with the possibility—or, rather, probability—that what at one time may be a comparatively harmless bacillus, may subsequently, in some unknown way, acquire malignant characters, and give rise to disease of the very gravest description."

The bearing of this on many questions in the general pathology of infectious disorders is very obvious. It appears that in the plague belt the disease is ever active now in one place, now in another, and it becomes a matter of the extremest interest to ascertain the relation of these two very different types of disease, and to discover the causes of the change in virulence of the bacillus on which they seem to depend.

In regard to the mode of infection, Dr. Lowson thinks that the popular idea that the disease becomes epidemic among rats before each epidemic among men is without foundation. He has not found the bacilli in the soil, but as they are present in the fæces, the upper layers of the soil may easily become infected. The infection would seem to be carried from the latrines, and be lodged on the dirty floors of the houses, the germs either rising as dust when the dirt is disturbed and being inhaled or swallowed, or entering by abrasions on the skin.

